# Evaluation of Bcl-2 Family Gene Expression in Hippocampus of 3, 4-methylenedioxymethamphetamine Treated Rats

**Published:** 2011-12-22

**Authors:** Sara Soleimani Asl, Mohammad Hassan Farhadi, Kazem Moosavizadeh, Ali Samadi Kuchak Saraei, Mansoure Soleimani, Seid Behnameldin Jamei, Mohammad Taghi Joghataei, Alireza Samzadeh-Kermani, Hamed Hashemi-Nasl, Mehdi Mehdizadeh

**Affiliations:** 1. Department of Anatomical Sciences, Hamadan University of Medical Sciences, Hamadan, Iran; 2. Substance Abuse and Dependence Research Center, University of Social Welfare and Rehabilitation Sciences, Tehran, Iran; 3. Cellular and Molecular Research Center, Tehran University of Medical Sciences, Tehran, Iran; 4. Department of Anatomical Sciences, Tehran University of Medical Sciences, Tehran, Iran; 5. Department of Chemistry, Faculty of Sciences, Zabol University, Zabol, Iran

**Keywords:** 3, 4-methylendioxymethamphetamine (MDMA), Apoptosis, Bcl-2, Bax

## Abstract

**Objective:**

3,4-methylenedioxymethamphetamine (MDMA) is an illicit, recreational drug that causes cellular death and neurotoxicity. This study evaluates the effects of different doses of MDMA on the expression of apoptosis–related proteins and genes in the hippocampus of adult rats.

**Materials and Methods:**

In this expremental study,a total of 20 male Sprague Dawley rats (200-250 g ) were treated with MDMA (0, 5, 10, 20 mg/kg i.p. twice daily) for 7 days. Seven days after the last administration of MDMA, the rats were killed. Bax and Bcl-2 genes in addition to protein expressions were detected by western blot and reverse transcriptionpolymerase chain reaction (RT-PCR).Results were analyzed using one-way ANOVA and p≤0.05 was considered statistically significant.

**Results:**

Our results showed that MDMA caused dose dependent up-regulation of Bax and down-regulation of Bcl-2 in the hippocampus. There was a significant alteration in bcl-2 and bax genes density.

**Conclusion:**

Changes in apoptosis-related proteins and respective genes relating to Bax and Bcl-2 might be involved in the molecular mechanism of MDMA-induced apoptosis.

## introduction

Previous experimental studies have shown that 3,4-methylenedioxymethamphetamine (MDMA)- induced neurotoxicity is characterized by functional impairment in memory and depression (1-3). MDMA causes acute release of 5-hydroxytryptamine (5-HT) from nerve endings (4), as well as destruction of 5-HT axons and 5-HT transporters (5) involved in the development of hyperthermic responses (6), and hyperactivity (7). Oxidative stress responses involve MDMAinduced neurotoxicity that lead to the formation of hydroxyl radicals (8), lipid peroxidation (9), and an increase in the number of Tunel positive cells in the hippocampus (10). MDMA can increase glial fibrillary acidic protein, an astrocyte protein that serves as a marker of injury-induced gliosis (11), and leads to cell death in the brain (12,13). Jimenezet al. has shown that MDMA induces cell death through an apoptotic pathway by releasing Cytochrome c (Cyt c) and activating the caspase cascade (14). MDMA treatment results in the decrease of intracellular Glutathione(GSH) and neural death (15). Another study has stated that MDMA can induce neural apoptosis and the expression of apoptosis-related factors, such as caspase 3 and Cyt c in rat brains (16).

The Upreti et al.study has shown that MDMA induces activation of c-Jun protein, N-terminal protein kinase, and p38 kinase that phosphorylates the anti-apoptotic Bcl-2 protein and promotes apoptosis in MDMA-exposed tissues (17). Apoptosis is a gene-regulated phenomenon that occurs under both physiological and pathological conditions. This mechanism is regulated by several sets of genes, the best characterized of which are the Bcl-2 family (18). The Bcl-2 family consists of anti-apoptotic (Bcl-2, Bcl-xL and Bcl-w) and proapoptotic (Bax, Bak, Bid and Bad) members (19). The cellular and molecular mechanisms involved in MDMA-induced neurotoxicity have not been fully elucidated. Since MDMA causes memory impairment, and because the hippocampus is an important structure that involves spatial memory, the main objective of this study is to elucidate the effects of different dosages of MDMA on the expression patterns of Bcl-2 and Bax in the hippocampus of male rats.

## Materials and Methods

The expremental study was carried out in accordance with a protocol approved by the Ethics Committee of Tehran University of Medical Sciences. All experiments were conducted to minimize both the number of animals used and the suffering caused by the procedures.

### MDMA preparation

MDMA was obtained from the Presidency Drug Control Headquarters. Solutions were made in sterile saline at a concentration such that each group received 1 ml/kg of the drug solution or only saline.

### Animals

A few studies have determined that there are sex differences in the pharmacokinetics following administration of MDMA in animals and humans. Males are more sensitive to acute toxicity than females (20, 21). This sensitivity may be due to differences in CYP1A2 activity and the N-demethylation pathway (22). In this study we have used male rats, as they are more sensitive to MDMA toxicity.

A total of 20 adult male Sprague Dawley rats of weights 200-250 g were obtained from Razi Institute. The rats were allowed to acclimatize to the colony room for 1 week prior to MDMA administration. Rats were maintained in the colony room at a temperature of 21 ± 1℃ (50% ± 10% humidity) on a 12 hours light/12 hours dark cycle with access to water and food ad libitum.

### MDMA administration

The 20 rats were assigned as follows. The control sham group (n =5) received normal saline (1 cc/kg, i.p.) twice daily for 1 week. The MDMA groups (n =15, 5 per dosage group) received either 5, 10, or 20 mg/kg MDMA (i.p.) twice daily for 1 week at 9:00 am and 5:00 pm (1, 3, 12). After the last administration, rats were maintained in the colony room for an additional week without any changes. Then, animals were killed by cervical dislocation, their brains were rapidly removed, and hippocampi were dissected out on ice, then frozen in liquid nitrogen and kept at -80℃ until analyzed.

### Western blot experiment

Immunoblot analysis was carried out with the hippocampi dissected from mice brains of MDMA and saline-treated rats. The frozen hippocampi were homogenized with ice-cold lysis buffer (that contained RIPA buffer with protease inhibitor cocktail, 1:10) for 1 hour and centrifuged (Eppendrof, Hamburg, Germany) at 12000 g for 20 minutes at a temperature of 4℃. The supernatant was removed and conserved. After determining the protein concentration with a Bio-Rad assay system (Bio-Rad, San Francisco, CA, USA), aliquots of 100 µg of protein from each sample were denatured with a sample buffer (6.205 mM Tris-HCl, 10% glycerol, 2% SDS, 0.01% bromophenol blue and 50 mM 2-ME) at 95℃ for 5 minutes and separated on 10% sodium dodecyl sulfate polyacrylamide gel electrophoresis (90 minutes, 120 voltage). Then, proteins were transferred to a Hybond-PTM membrane (Amersham Pharmacia Biotech, Piscataway, New Jersey, USA). Membranes were blocked with 5% nonfat milk dissolved in TTBS buffer (Tris 50 mM, NaCl 1.5%, and Tween 20 0.05%, pH =7.5) for 1 hour. Nitrocellulose membranes were stained withanti-Bcl-2 and anti-Bax monoclonal antibodies (1:1000 Sigma Aldrich, St. Louis, MO, USA) for 2 hours, followed by secondary antibody alkaline phosphatase-conjugated anti-mouse antibodies (1:10000, Sigma Aldrich, St. Louis, MO, USA) for 1 hour. Bands were detected by the chromogenic substrate, 5-bromo-4-chloro-3 -indolyl phosphate, in the presence of nitroblue tetrazolium. β-actin antibody (1:1000, Sigma Aldrich, St. Louis, MO, USA) was used to detect the endogenous standard for normalization. The bands from various groups that corresponded to the appropriate molecular weight for each subunit were analyzed and values were compared by densitometric measurements, using an image analysis system (UVIdoc, Houston, Texas, USA).

### RNA extraction and detection of gene products by reverse transcription-polymerase chain reaction

Total mRNA was extracted from the hippocampi by a phenol-chloroform extract. Tissue samples were homogenized in 1000 µl RNATM (Cinnagen, Tehran, Iran) followed by the addition of 200 µl ice-cold chloroform. The homogenates were centrifuged (Eppendrof, Hamburg, Germany) at 12000 g for 20 minutes at 4℃. The RNA of the water-soluble supernatant was precipitated with isopropanol and washed with 75% ethanol. The air-dried RNA pellet was dissolved in RNase-free water. cDNA first-strand synthesis was performed by a cDNA synthesis kit (Quiagen, Hilden, Germany) following the protocol outlined by the company. First strand cDNA (2.5 ml) was used as a template for subsequent PCR with a PCR master kit (Cinnagen, Tehran, Iran) and corresponding primers (Cinnagen, Tehran, Iran; Table 1).

For PCR, 1 µl of cDNA was placed into 24 µl of reaction volume that contained 12.5 µl Master Mix, 1 µl of each primer, and 9.5 µl sterile deionized water. The PCR reactions included initial denaturation at 95℃ for 3 minutes followed by 31 cycles at 95℃ for 20 seconds, 65℃ for 30 seconds, and 72℃ for 30 seconds for Bax. For Bcl-2, it was 35 cycles at 95℃ for 30 seconds, 60℃ for 1 minute, and 72℃ for 60 minutes. The reactions were terminated by an elongation period at 72℃ for 7 minutes. The same annealing temperature was used for beta-actin. PCR products were separated by electrophoresis in 1.5% agarose gel at 100 V. Semi-quantitative analyses were assessed using a digital imaging system (UVIdoc, Houston, Texas, USA).

### Statistical analysis

Data were presented as mean ± SEM. The results were analyzed by one-way ANOVA and post-hoc comparisons were performed using the Tukey test. P≤0.05 was considered statistically significant.

## Results

### Effect of MDMA on pro-apoptotic Bax protein and gene expression

**Fig 1:**
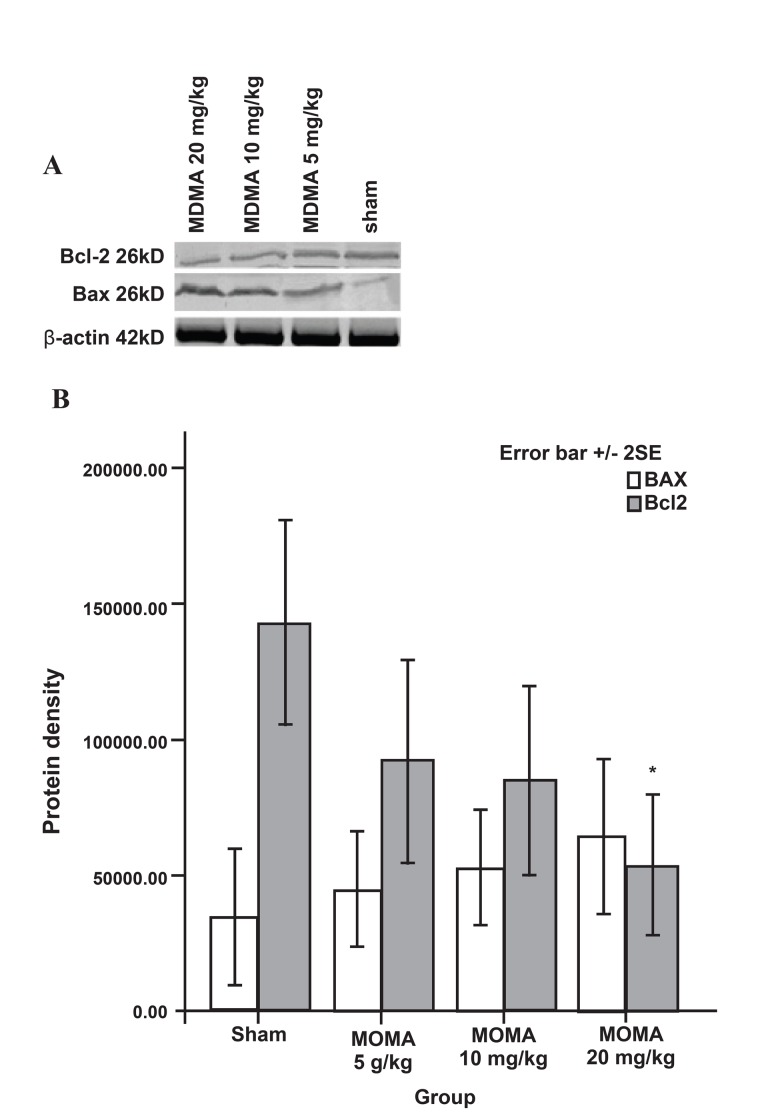
Western blot analysis of Bax and Bcl-2 protein expressions in sham and **MDMA** groups (n=5 per group) that received **MDMA** (0, 5, 10, 20 mg/kg i.p. twice daily) for 7 days. The frozen hippocampi were lysed, transferred to nitrocellulose paper, incubated with anti-Bcl-2, anti-Bax antibodies, and secondary anti-mouse antibody. Bands were then detected by chromogenic substrate. Data were analyzed using one-way analysis of variance (**ANOVA**) followed by Tukey test for multiple comparisons. *p<0.001 vs. sham group.

MDMA caused both the up-regulation of the pro-death Bax protein and gene expression. MDMA caused a dose-dependent increase in Bax protein expression. High doses of MDMA lead to increased expression of Bax protein as follows: for the control, the mean expression was 346.1 ± 1.25; whereas it was 449.37 ± 1.06 for 5 mg/kg; 525.62 ± 1.07 for 10 mg/kg; and 639 ± 1.43 for 20 mg/kg of MDMA. However, the results were not statistically significant (Fig 1).

**Fig 2:**
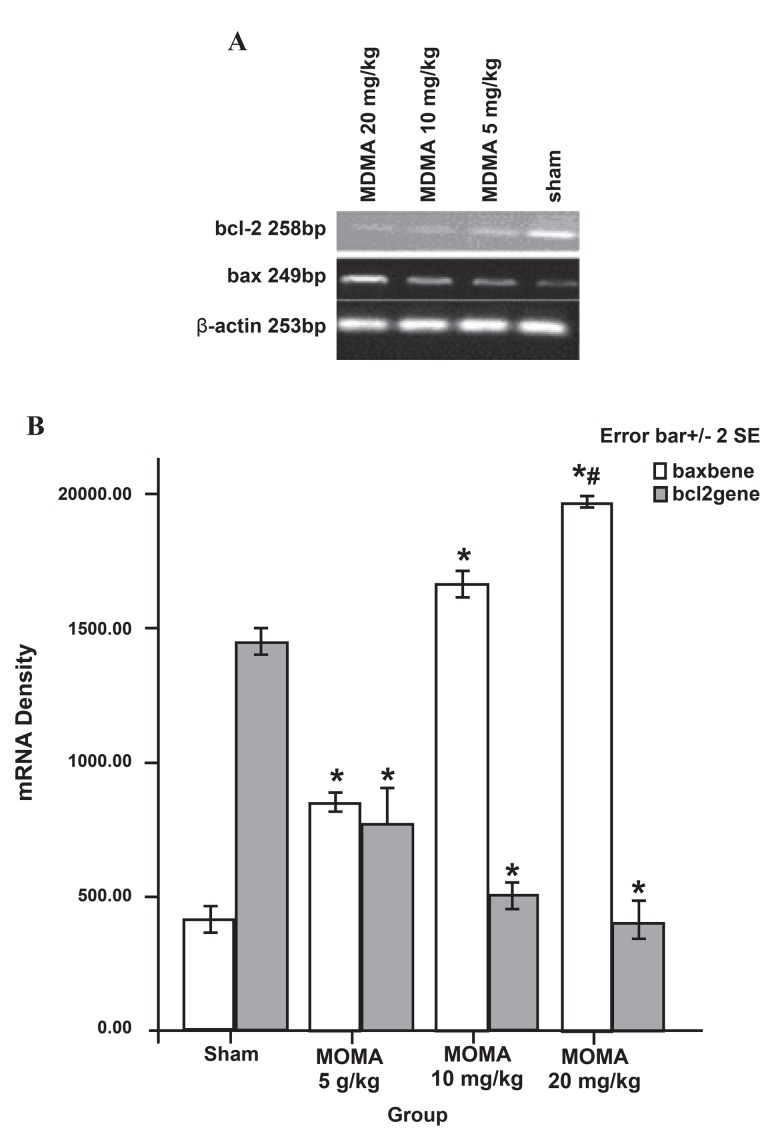
bax and bcl-2 gene expression in sham and **MDMA** groups (n =5 per group) that received **MDMA** (0, 5, 10, 20 mg/ kg i.p. twice daily) for 7 days. Total **RNA** was extracted using phenol-chloroform. After synthesis of c**DNA**, **PCR** was performed with the respective primers. Data were analyzed using one-way analysis of variance (**ANOVA**) followed by Tukey’s test for multiple comparisons. *p<0.001 vs. sham group, p<0.001 vs.5 mg/kg and 10 mg/kg groups.

Regarding bax mRNA expression, there was a significant difference between the MDMA treatment groups compared to the saline control groups (p<0.001, Fig 2). There was a significant difference between the MDMA groups (mean: 409.6 ± 22.49 for the saline control; 848.8 ± 15.21 for the 5 mg/kg group, 1655.0 ± 24.79 for 10 mg/kg, and 1967.2 ± 7.91 for 20 mg/kg group of MDMA. Thus, the bax gene was more significantly expressed in the 20 mg/kg MDMA group compared to the 5 and 10 mg/kg groups (p<0.001, Fig 2).

**Table 1:** Primers for beta-actin, bax, and bcl-2 genes


Genes	Forward primer	Reverse primer
beta-actin	5'-TGGAGAAGAGCTATGAGCTGCCTG-3'	5'-GTGCCACCAGACAGCACTGTGTTG-3'
bax	5'-CCAAGAAGCTGAGCGAGTGTCTC-3'	5'-AGTTGCCATCAGCAAACATGTCA-3'
bcl-2	5'- CGCCCGCTGTGCACCGAGA -3'	5'-CACAATCCTCCCCCAGTTCACC-3'


### Effect of MDMA on anti-apoptotic Bcl-2 protein and gene expression

MDMA caused a dose-dependent decrease in Bcl-2 protein expression in comparison to the saline control group (mean: 142.76 ± 1.89 for the saline control; 91.9 ± 1.85 for the 5 mg/kg group; 85.03 ± 1.72 for the 10 mg/kg; and 53.61 ± 1.29 for 20 mg/kg MDMA). Significant difference was noted in the 20 mg/kg MDMA group when compared to the saline control group (p<0.01, Fig 1). In contrast to the prodeath bax gene, bcl-2 gene expression decreased in MDMA groups when compared to the saline control groups (mean: 1440.2 ± 27.28 for the saline control; 768 ± 63.32 for 5 mg/kg; 502.00 ± 20.14 for the 10 mg/kg; and 402.02 ± 34.84 for the 20 mg/ kg MDMA groups; p<0.001, Fig 2). There was a significant difference between the 10 and 20 mg/kg groups and the 5 mg/kg group (p<0.001, Fig 2).

## Discussion

Our previous study has shown that MDMA causes learning memory impairment in the Morris water maze test (1). The hippocampus is one of the most important brain structures associated with learning memory and cognition (23). Hippocampus neurons can be damaged by many neurotoxic factors, such as drugs and diseases (24, 25). MDMA as a psychostimulant drug can induce learning memory and impair hippocampal mental function (3,26). In this study, we have focused our efforts on the MDMA-induced apoptosis mechanism in the hippocampus. The results of this research demonstrated that MDMA led to up-regulation of the bax gene and down-regulation of the bcl-2 gene, and subsequently their proteins (Figs 1, 2) in the rat hippocampus.

These results are consistent with a study by Jayanthi that showed an injection of methamphetamine as another amphetamine derivative triggers the activation of the programmed cell death pathway in mammals. They have demonstrated that methamphetamine causes up-regulation of Bax and down regulation of Bcl-2 proteins (27). The Montagomy et al. study has shown that MDMA causes rapid intracellular Ca2+ influx, mitochondrial membrane depolarization, ROS production, and caspase-dependent DNA fragmentation (28).

It has been described that MDMA metabolites can increase the production of reactive species and protein-bound quinines (both dose and timedependently), deplete intracellular glutathione, induce oxidative stress, and neural death (15).

Reizzo et al. have shown that injection of a single dose of MDMA in rats caused an increase in oxidative stress and subsequent apoptosis (TUNEL assay) in the hippocampus, striatum, and frontal cortex (10). Treatment with MDMA or METH caused an increase in the number of cells with chromatin condensation, DNA fragmentation, and a significant increase in caspase-3 activity and Cyt c release as markers of apoptosis (14). These studies were consistent with our present study, which has shown that MDMA causes up-regulation of Bax and down-regulation of Bcl-2. The Bcl-2 family of proteins regulates mitochondrial changes such as PT pore (a poly protein channel) and Cyt c release. Bax interacts with PT pore, causing conformational changes and Cyt c release (29).

## Conclusion

Our observations suggest that multiple doses of MDMA can induce cell death through an apoptotic pathway, implicating up-regulation of Bax and downregulation of Bcl-2. We have also shown that nonacute apoptotic effects of this drug are dose-dependent. To prove the anti-apoptosis effects of MDMA, more assays on the caspase family and other members of the Bcl-2 family need to be performed.
